# Extrinsic Origin of Persistent Photoconductivity in Monolayer MoS_2_ Field Effect Transistors

**DOI:** 10.1038/srep11472

**Published:** 2015-06-26

**Authors:** Yueh-Chun Wu, Cheng-Hua Liu, Shao-Yu Chen, Fu-Yu Shih, Po-Hsun Ho, Chun-Wei Chen, Chi-Te Liang, Wei-Hua Wang

**Affiliations:** 1 Institute of Atomic and Molecular Sciences, Academia Sinica, Taipei 106, Taiwan; 2 Department of Physics, National Taiwan University, Taipei 106, Taiwan; 3 Department of Materials Science and Engineering, National Taiwan University, Taipei 106, Taiwan

## Abstract

Recent discoveries of the photoresponse of molybdenum disulfide (MoS_2_) have shown the considerable potential of these two-dimensional transition metal dichalcogenides for optoelectronic applications. Among the various types of photoresponses of MoS_2_, persistent photoconductivity (PPC) at different levels has been reported. However, a detailed study of the PPC effect and its mechanism in MoS_2_ is still not available, despite the importance of this effect on the photoresponse of the material. Here, we present a systematic study of the PPC effect in monolayer MoS_2_ and conclude that the effect can be attributed to random localized potential fluctuations in the devices. Notably, the potential fluctuations originate from extrinsic sources based on the substrate effect of the PPC. Moreover, we point out a correlation between the PPC effect in MoS_2_ and the percolation transport behavior of MoS_2_. We demonstrate a unique and efficient means of controlling the PPC effect in monolayer MoS_2_, which may offer novel functionalities for MoS_2_-based optoelectronic applications in the future.

Following the discovery of graphene^1^,^2^,^3^, two-dimensional (2D) materials have emerged as one of the most important research topics in condensed matter physics because of the novel phenomena exhibited by and the promising applications of these materials^4^,^5^,^6^. In particular, semiconducting layered materials^7^,^8^, such as transition metal dichalcogenides (TMD)^9^,^10^, can complement graphene because of their intrinsic bandgap and therefore can enrich the properties of these 2D materials^11^,^12^. Molybdenum disulfide (MoS_2_) is a layered semiconducting TMD and therefore exhibits a bandgap^13^,^14^, high mobility^15^,^16^ and strong mechanical properties^17^. Moreover, unique physical properties, including spin-valley coupling and the layer dependence of the band structure in MoS_2_, have been demonstrated^18^,^19^,^20^. The combination of these interesting properties has made MoS_2_ very attractive for new functionalities such as sensors^21^,^22^,^23^, logic circuits^24^,^25^ and optoelectronic devices^26^,^27^,^28^,^29^,^30^.

Recently, high photoresponsivity^31^,^32^, the photovoltaic effect^29^ and the photothermoelectric effect^28^. have been reported in monolayer MoS_2_-based photodetectors and phototransistors. Optoelectronic studies on these MoS_2_ devices demonstrated persistent photoconductivity (PPC), which is sustained conductivity after illumination is terminated^32^,^33^,^34^,^35^. However, a detailed understanding of PPC and its mechanism in MoS_2_ is still not available. The origin of the trap states in MoS_2_, which lead to the PPC effect, remains under debate^36^,^37^,^38^,^39^. Moreover, the PPC effect modifies the transport properties of MoS_2_ samples, which are sensitive to the history of photon irradiation. Therefore, it is essential to understand the PPC effect to control transport phenomena in MoS_2_.

Here, we present a systematic study of PPC in monolayer MoS_2_ field effect transistors. The PPC dependence on the temperature, the photon dose, and the excitation energy enabled us to attribute the PPC in MoS_2_ to random localized potential fluctuations that hinder the recombination of photoexcited electron-hole pairs. Comparing the PPC in suspended and substrate-supported MoS_2_ devices led us to conclude that PPC originates primarily from extrinsic sources. Moreover, we could correlate PPC phenomena with percolation transport, whereby carriers transfer among nearest low-potential puddles.

Details on the fabrication of the MoS_2_ field effect transistors can be found in the Supporting Information S1. In brief, we mechanically exfoliated MoS_2_ flakes onto an octadecyltrichlorosilane (OTS)-functionalized SiO_2_/Si substrate, thus obtaining a hydrophobic surface that minimized the amount of charged impurities in the 2D materials^40^. Monolayer MoS_2_ flakes were identified by optical microscopy and subsequently confirmed using Raman spectroscopy and photoluminescence (PL) measurements (Supporting Information S1). We deposited electrical contacts (Au, 50 nm) in a two-probe geometry using a residue-free approach^41^,^42^ to minimize contamination from the conventional lithography process. The MoS_2_ samples were stored under vacuum for 12 hours before performing the measurements to reduce the number of adsorbed molecules (Supporting Information S2). This procedure enabled us to investigate the photoresponse of MoS_2_ without interference from the gas adsorbate effect^35^.

## Results and Discussion

**Figure 1:**
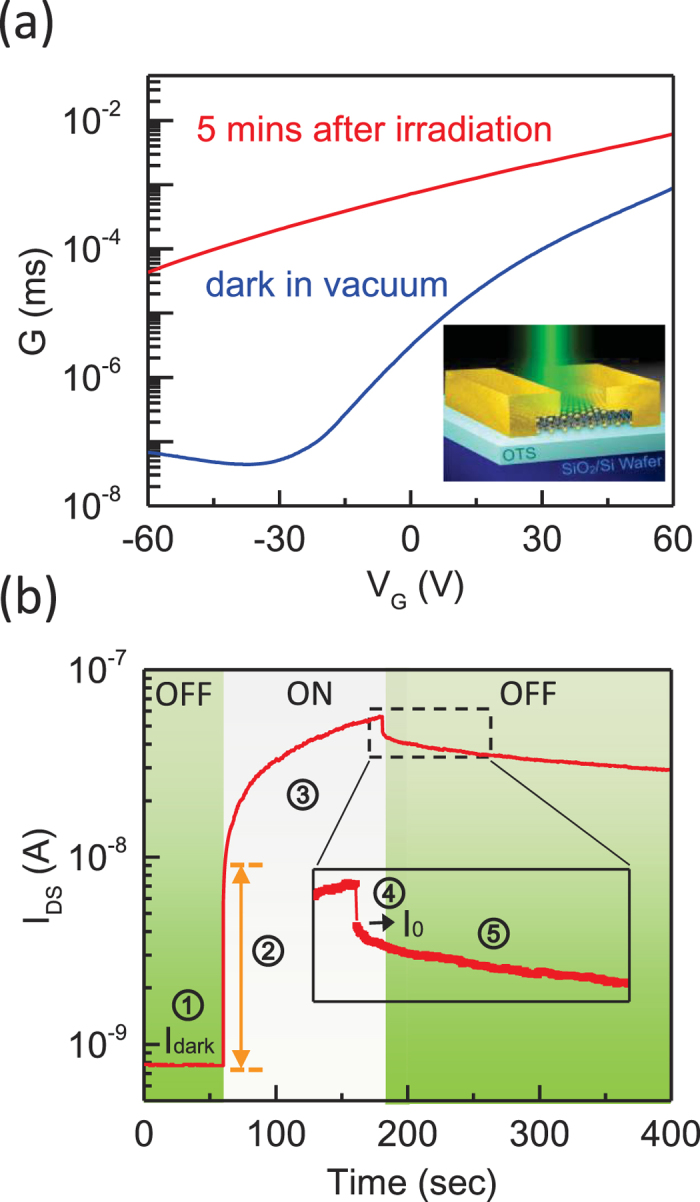
The PPC effect in a monolayer MoS_2_ transistor. (**a**) Transconductance as a function of *V*_*G*_ in the dark and after illumination at room temperature in vacuum, showing that the conductance of the device is greatly enhanced after illumination and remains in a high-conductivity state for a long period of time. Inset: a schematic of a MoS_2_ device on an OTS-functionalized substrate. (**b**) The photoresponse of the MoS_2_ device for *V*_*G*_ = 0V and *V*_*DS*_ = 50 mV, which can be classified into 5 stages. In addition to the photoresponse due to band-to-band transition (stages 2 and 4), the device exhibits a slow increase in the photocurrent under illumination (stage 3) and the PPC effect (stage 5).

Figure 1a shows the two-probe transconductance of sample A as a function of the back-gate voltage (*G* − *V*_G_). The MoS_2_ device exhibited typical n-type channel characters with a mobility of 0.7 cm^2^/Vs and an on/off ratio of 2 × 10^4^. The MoS_2_ transistor was then illuminated by a laser (wavelength = 532 nm) with a spot size of ≃1.5 μm. After the illumination was terminated, the conductance of the device was greatly enhanced and remained in a high-conductivity state for a time period from 2 minutes to 2 hours, depending on the irradiated photo-dose. It is noted that the longer the illumination time, the higher was the conductance.

We subsequently investigated the PPC in the MoS_2_ transistor in greater detail. Figure 1b shows the temporal evolution of the source–drain current (*I*_*DS*_ − *t*) in vacuum (Supporting Information S3). The fast response of the current (stage 2) was attributed to a band-to-band transition that created conducting electrons and holes^26^,^27^. After the initial upsurge from the dark current (*I*_*dark*_) level, the *I*_*SD*_ increased gradually to over 2 orders of magnitude (stage 3) above the dark current. This slow increase in the *I*_*SD*_ could not be attributed to the common band-to-band transition. After the laser irradiation was terminated, the *I*_*SD*_ exhibited a rapid drop because of the band-to-band transition (stage 4), followed by noticeable PPC (stage 5). The photocurrent at which PPC began to dominate is denoted by *I*_*0*_. This PPC effect was consistently observed in all of our 10 MoS_2_ devices. For MoS_2_ under ambient conditions, a short photoresponse time below 1 sec has been previously reported^26^ and attributed to the presence of gas adsorbates^35^. However, adsorbates were not a significant factor in the present study because our MoS_2_ samples were treated in vacuum.

**Figure 2:**
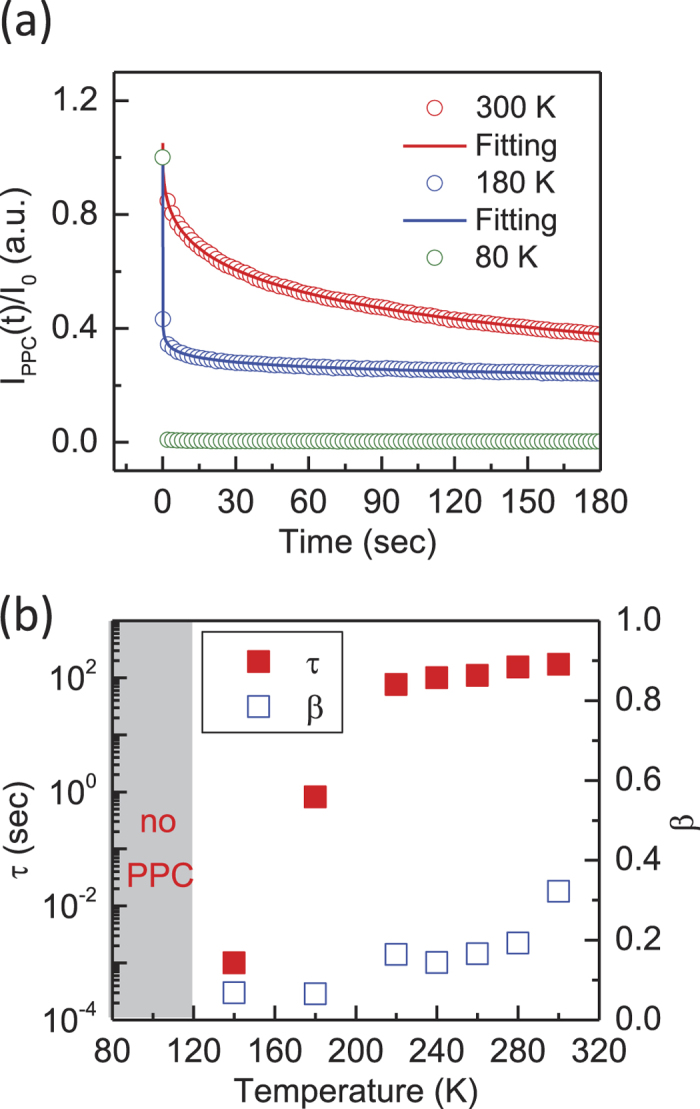
The temperature dependence of PPC. (**a**) Three characteristic PPC relaxations (circles) at *T* = 80, 180, and 300 K. PPC is more pronounced at higher temperatures but is greatly suppressed at *T* = 80 K. The PPC relaxations are well described by a stretched exponential decay (solid line). The stretched exponential decay is *I*_*PPC*_(*t*) = *I*_o_exp[ – (*t/τ*)^*β*^] with the dark current subtracted. *I*_*0*_ in the PPC relaxation curves is normalized to 1 for comparison of the decay rate. (**b**) The temperature dependence of the decay time constant (*τ*) and the exponent (*β*). The grey region indicates the temperature range over which PPC is not observed.

We first discuss the temperature dependence of PPC in the MoS_2_ devices, which provides important indications of the PPC mechanism^43^. Figure 2a is a comparison of three characteristic PPC relaxations for sample A at *T* = 80, 180, and 300 K. For purposes of comparison, the dark level was subtracted, and the PPC was normalized by *I*_*0*_. It is noted that the PPC was more pronounced at high temperatures, although the photocurrent relaxed very fast and the PPC was considerably weakened at low temperatures (Supporting Information S4). The temperature dependence of the PPC was measured by heating the samples up to room temperature, allowing the carriers to relax to equilibrium^38^,^44^, and then cooling the samples down in the dark to the target temperature. The PPC relaxation curves were well-fitted by a single stretched exponential decay^45^,
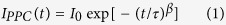
where *τ* is the decay time constant, and *β* is the exponent (Supporting Information S5). The stretched exponential decay has been widely used to model relaxation processes in complex and slowly relaxing materials^43^,^44^,^45^,^46^. Therefore, this photocurrent decay suggests that PPC is related to disorders in the MoS_2_ devices.

The fitted *τ* and *β* values at different temperatures are shown in Fig. 2b. Clearly, the MoS_2_ device exhibited a shorter *τ* at low temperatures, which is a signature of the random local potential fluctuations (RLPF) model^43^,^44^. In the RLPF model, local potential fluctuations arise either because of intrinsic disorders in the materials or extrinsic charged impurities. Therefore, low-energy electrons and holes become localized in potential minima and are spatially separated, resulting in a long recombination lifetime. The thermal excitation of carriers to higher energy states above the mobility edge produces a photocurrent after the irradiation is terminated, resulting in the PPC effect. At low temperature, the carriers are well-confined inside charge traps and therefore, negligible PPC is observed. This RLPF model has been widely used to explain PPC in II-VI compound semiconductors^43^,^44^,^46^. However, this model has not been used to explain the PPC effect in 2D TMD materials.

**Figure 3:**
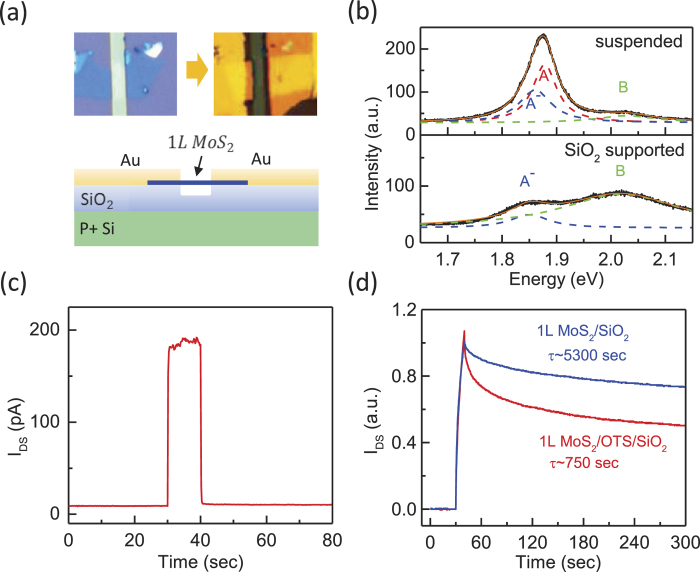
The substrate effect of PPC (**a**) Upper panel: optical images of the suspended MoS_2_ device before and after deposition of the electrode. Lower panel: a schematic of the suspended monolayer MoS_2_ device. (**b**) PL spectra of suspended (upper panel) and SiO_2_-supported (lower panel) monolayer MoS_2_ devices. (**c**) Photoresponse of the suspended MoS_2_ device showing negligible PPC at *T* = 300 K (*V*_*G*_ = 0V, *V*_*DS*_ = 5V). (d) Photoresponses of monolayer MoS_2_ on OTS-functionalized and conventional SiO_2_ substrates (*V*_*G*_ = 0V, *V*_*DS*_ = 5 V). The photocurrents are normalized by *I*_*0*_ for purposes of comparison.

The PPC in the MoS_2_ samples was attributed to the RLPF model in which the potential fluctuations originate from extrinsic^36^,^47^ or intrinsic^48^,^49^ sources. Extrinsic adsorbates or chemical impurities in the vicinity of the MoS_2_ samples can lead to trap states^36^,^47^. Alternatively, sulfur vacancies^37^ and the formation of MoO_3_ in bulk MoS_2_ have intrinsic causes. We determined the origin of the potential fluctuations by investigating the substrate effect on the PPC. We first measured the PPC in suspended MoS_2_ devices that were fabricated by exfoliating monolayer MoS_2_ onto SiO_2_/Si substrates with trenches (width ≃ 2 μm). Figure 3a shows the optical images of a fabricated suspended MoS_2_ device before and after deposition of the electrodes. To verify the suspension of the MoS_2_ sample, we measured the PL spectra of the sample, which are very sensitive to the existence of SiO_2_/Si substrates^50^. Figure 3b is a comparison of the PL spectra of a suspended MoS_2_ device (sample B) and a SiO_2_-supported MoS_2_. The PL spectrum of the suspended MoS_2_ exhibited a strong exciton peak (A), along with trion (A^-^) and exciton (B) peaks. The presence of the pronounced exciton peak A indicated that the MoS_2_ sample was free from the strong n-type doping effect of the SiO_2_ substrate^50^, indicating a suspended structure. In contrast, only trion (A^-^) and exciton (B) peaks were observed in the PL spectrum of SiO_2_-supported MoS_2_.

Next, we discuss the photoresponse of the suspended MoS_2_ (sample B) that is shown in Fig. 3c. Interestingly, sample B exhibited a negligible PPC effect at *T* = 300 K, in contrast to the photoresponse of the substrate-supported MoS_2_ at the same temperature. Only the photoresponse from the band-to-band transition was observed in this suspended MoS_2_ device. The absence of the PPC effect in this control sample clearly indicated that the potential fluctuations in our MoS_2_ devices had extrinsic sources. We also compared the PPC effect for MoS_2_ devices that were fabricated on an OTS-functionalized SiO_2_ surface and those that were fabricated on conventional SiO_2_ substrates, as shown in Fig. 3d. The PPC effect in the MoS_2_/SiO_2_ device was stronger than that in the MoS_2_/OTS/SiO_2_ device. This substrate effect was consistently observed in several samples, suggesting that there were fewer charge traps in the MoS_2_/OTS/SiO_2_ devices than in the MoS_2_/SiO_2_ device. This difference was reasonable because the OTS-functionalized SiO_2_ surface is known to be hydrophobic and could therefore reduce surface adsorbates^40^, resulting in smaller potential variations. Further study is required to identify these extrinsic sources in detail, e.g., gas adsorbates and/or chemical impurities on the SiO_2_ interface. Nevertheless, our finding demonstrates the importance of extrinsic sources, thereby providing a means of eliminating the PPC effect to control the photoresponse in TMD materials.

**Figure 4:**
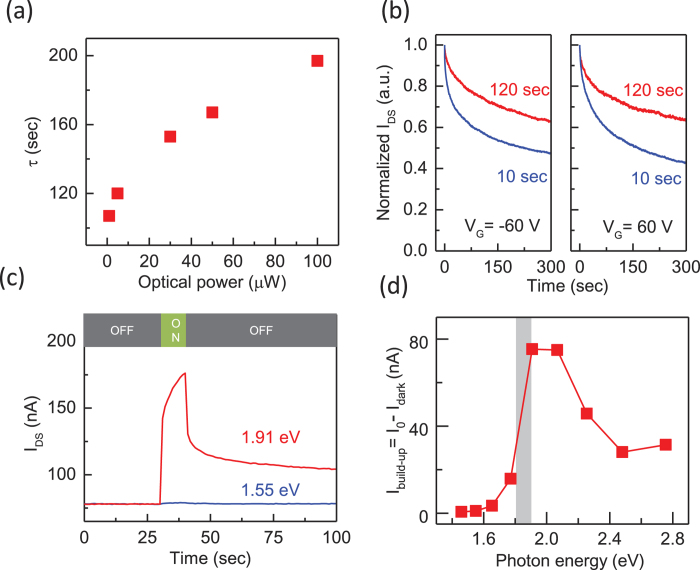
Photon dose and excitation wavelength dependences of the PPC relaxation. (**a**) Excitation power dependence of the decay time constant for *V*_*G*_ = –60 V and *V*_*DS*_ = 50 mV. (**b**) The PPC relaxations for different illumination times (10 seconds and 120 seconds) at *V*_*G*_ = ±60 V and *V*_*DS*_=50mV. (**c**) The temporal evolution of *I*_*SD*_ for excitation energies below (*E*_*ex*_ = 1.55 eV) and above (*E*_*ex*_ = 1.91 eV) the bandgap at *T* = 300 K and *V*_*G*_ = 0V. (**d**) The PPC build-up level (*I*_*build-up*_) at different excited photon energies and *V*_*G*_ = 0V. The photon dose and excitation energy dependences of the PPC are consistent with the RLPF model in the monolayer MoS_2_ devices.

Next, we present the PPC dependence on the photon dose and the excitation energy to further validate the RLPF mechanism in our MoS_2_ samples. Figure 4a shows the excitation power dependence of *τ* at room temperature, where the PPC increases with the excitation power. In the RLPF model, more carriers are excited under higher photon doses, and more electrons and holes can redistribute to occupy the sites of the local potential minima. This redistribution of carriers is therefore enhanced under high excitation power, resulting in a larger *τ* after the photoexcitation is terminated^46^. Figure 4b shows the PPC relaxation for different illumination times at room temperature, where *τ* increases with the illumination time (Supporting Information S6), Similar to the effect of excitation power, this PPC dependence on the photon dose in MoS_2_ devices is in good agreement with the RLPF mechanism.

We further investigated the PPC of monolayer MoS_2_ at different excitation energies ranging from 1.46 eV to 2.75 eV (Supporting Information S3), which included the optical bandgap in monolayer MoS_2_ ≃1.8 eV^13^,^14^. Figure 4c is a comparison of the temporal evolution of the photoresponse for excitation energies below (*E*_*ex*_ *=* 1.55 eV) and above (*E*_*ex*_ *=* 1.91 eV) the bandgap. While the *I*_*DS*_ − *t* curve for *E*_*ex*_ *=* 1.91 eV exhibited a typical photoresponse (which is similar to that in Fig. 1b), the photoresponse was insignificant for *E*_*ex*_ *=* 1.55 eV. Figure 4d shows the excitation energy dependence of the PPC build-up level (*I*_*build-up*_), which is defined as *I*_*0*_*–I*_dark_. Here, *I*_*build-up*_ represents the charging process in which photoexcited carriers fill up the local potential minimum during illumination. The photoresponse was clearly activated at *E*_*ex*_ ~ 1.8 eV, showing that the bandgap of monolayer MoS_2_ is related to the PPC^51^. In the RLPF model, *I*_*build-up*_ is initiated by the photoexcited carriers through the band-to-band transition, which are subsequently confined by the trap states. Therefore, PPC can only occur when the photon energy surpasses the bandgap. To briefly summarize, our observations of the PPC dependence on the temperature, the substrate effect, the photon dose, and the excitation energy fully support the RLPF model as the mechanism of PPC in the monolayer MoS_2_ devices.

In addition to RLPF, two other mechanisms, large lattice relaxation (LLR)^52^,^53^ and the microscopic barrier (MB)^54^,^55^, are well-known mechanisms for PPC in a variety of materials, including mixed crystals, semiconductors, and heterostructures. In the LLR model, electrons are photoexcited from deep-level traps, and an energy barrier prevents the recapture of the electrons^52^,^56^, resulting in the PPC effect. Because the recapture is a thermally activated process, the PPC due to LLR is more pronounced at low temperatures, which is inconsistent with our observations (Fig. 2). Another feature of the PPC that results from LLR is that a photocurrent can be activated by excitation from deep-level traps to conduction bands^57^ with energies below that of the bandgap, which also contradicts the observed dependence of the PPC on the excitation energy. The MB is another mechanism for PPC in which photoexcited electron–hole pairs are spatially separated by a macroscopic potential barrier, followed by charge accumulation or trapping by barriers/spacers^54^,^55^. Recent studies on various structures, including MoS_2_/graphene^30^, quantum-dot/graphene^58^ and chlorophyll/graphene^59^ heterostructures, demonstrated noticeable PPC due to this MB model. However, there were no such artificially created structures in our MoS_2_ devices that could yield a macroscopic potential barrier. The PPC effect in the MB model also follows a single-exponential decay^54^, unlike the stretched exponential decay that was observed in our samples. We therefore conclude from our experimental data that LLR and MB are not mechanisms for PPC in our MoS_2_ devices.

**Figure 5:**
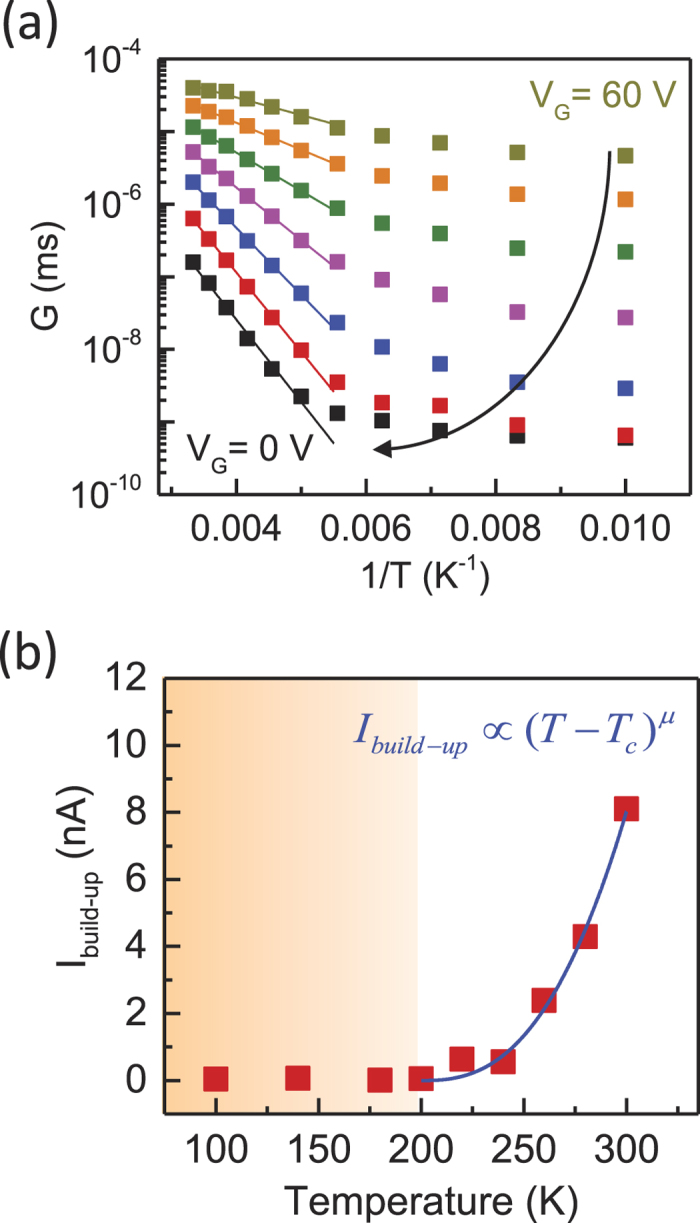
Correlations between PPC and transport behaviors. (**a**) The Arrhenius plot for the conductance of sample C at different *V*_*G*_ values. The solid lines indicate linear fits in the thermally activated regime. (**b**) The temperature dependence of the PPC buildup level (*I*_*build-up*_) at *V*_*G*_ = 0V. For *T > *200 K, *I*_*build-up*_ is well described by a percolation model, which is shown as a solid line. The shaded region denotes the temperature regime over which transport is dominated by tunneling between localized states.

Finally, we consider the connection between the PPC effect and the transport properties of the MoS_2_ devices. Figure 5a is an Arrhenius plot of the conductance of sample C at different 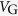 values and shows that insulating behavior (*dG/dT* > *0*) was found over the temperature range 80 < *T* *<* 300 K. For *T* *>* 200 K, the MoS_2_ sample exhibited thermally activated behavior, where the activation energy was extracted from the linear fit (*E*_a_ = 98 meV at *V*_G_ = 0 V). The carrier transport in this temperature regime could be described by a percolation model, in which conduction occurs via a network of spatially distributed charge puddles^60^. The activation energy then corresponded to the average potential barrier of the charge puddles. For 80 K *<* *T* *<* 200 K, the correlation of the puddles decreased because of the decrease in the thermal energy, and the carriers could only conduct by tunneling between localized states^60^. Therefore, there was a phase transition from localized to percolation transport. The transition temperature of sample C was *T*_*C*_ ~ 200 K, which corresponded to the temperature at which the *G*–1/*T* curve deviated from thermally activated behavior (more details are provided in Supporting Information S7). To compare the temperature dependence of the PPC and transport properties, we plot *I*_*build-up*_ as a function of temperature in Fig. 5b. The PPC is insignificant for T < 200 K because of the low conductivity in the localized transport regime. Notably, *I*_*build-up*_ exhibited an activated behavior for *T* *>* 200 K, where the transition temperature coincided with the *T*_*C*_ value that was extracted from the transport behavior. *I*_*build-up*_ could be described by a percolation approach^43^:
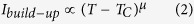
where *μ* is the characteristic exponent. We found that this function fits the data reasonably well (*μ* *=* *2.6*), indicating that the PPC relaxation was consistent with the percolation transport picture.

In conclusion, we have demonstrated that PPC in monolayer MoS_2_ can be controlled by temperature, the photon dose, the excitation energy, and the substrate effect. These characteristics show that PPC in MoS_2_ can be well-explained by the RLPF model. The potential fluctuations could be attributed to extrinsic sources because of the absence of PPC in the suspended MoS_2_ devices. Moreover, the temperature dependence of the PPC could be described as a transition from localization to percolation models, which was in agreement with the transport properties of MoS_2_. The results of this study can provide insight into PPC phenomena in monolayer MoS_2_, which is important for the development of MoS_2_-based optoelectronic applications.

## Methods

### Sample preparation

MoS_2_ flakes (SPI Supplies) were mechanically exfoliated onto octadecyltrichlorosilane (OTS) self-assembled monolayer (SAM) functionalized SiO_2_ (300 nm)/Si substrates. The surface of the OTS-functionalized SiO_2_/Si substrate was hydrophobic with a typical contact angle above 110°. First, the MoS_2_ flakes were identified and characterized under an optical microscope using variations in contrast and then were examined by Raman and photoluminescence spectra. We adopted resist-free fabrication to prevent contamination of the MoS_2_ samples from the resist residue of the conventional lithography process. We used nanowire as a shadow mask to deposit metallic contacts (Au, 50 nm) with an electron-beam evaporator at a base pressure of 1.0 × 10^–7^ Torr. The MoS_2_ devices were then transferred into a cryostat (Janis Research Company, ST-500) for electrical and optical characterization. The samples were stored under a high vacuum of 1 × 10^–6^ Torr to minimize the undesirable adsorption of chemical substances. A Keithley 237 was used to perform the DC electrical measurements, and a Keithley 2400 was used to apply the back gate voltage.

## Additional Information

**How to cite this article**: Wu, Y.-C. *et al*. Extrinsic Origin of Persistent Photoconductivity in Monolayer MoS_2_ Field Effect Transistors. *Sci. Rep*. **5**, 11472; doi: 10.1038/srep11472 (2015).

## Supplementary Material

Supplementary Information
