# Supplementary material for: Extrinsic Origin of Persistent Photoconductivity in Monolayer MoS2 Field Effect Transistors
Source: Sci Rep. 2015 Jun 26;5:11472. doi: 10.1038/srep11472 (PMC4650635; doi:10.1038/srep11472)
Supplement: Supplementary Information [file srep11472-s1.pdf]

Supporting Information

Extrinsic Origin of Persistent Photoconductivity in Monolayer MoS_2 Field Effect Transistors

Yueh-Chun Wu[1‡], Cheng-Hua Liu[1,2‡], Shao-Yu Chen[1§], Fu-Yu Shih[1,2], Po-Hsun Ho[3], Chun-Wei Chen[3],Chi-Te Liang[2], and Wei-Hua Wang[1*]

[1]Institute of Atomic and Molecular Sciences, Academia Sinica, Taipei 106, Taiwan

[2]Department of Physics, National Taiwan University, Taipei 106, Taiwan

[3]Department of Materials Science and Engineering, National Taiwan University, Taipei 106, Taiwan

[§]Current address: Department of Physics, University of Massachusetts, Amherst, Massachusetts 01003, United States

[‡]These authors contributed equally to this work.

[*]Corresponding Author. (W.-H. Wang) Tel: +886-2-2366-8208, Fax: +886-2-2362-0200;

S1. Device Fabrication

MoS_2 flakes (SPI Supplies) were mechanically exfoliated onto octadecyltrichlorosilane (OTS) self-assembled monolayer (SAM) functionalized SiO_2 (300 nm)/Si substrates. The surface of the OTS-functionalized SiO_2/Si substrate was hydrophobic with a typical contact angle above 110°. The hydrophobic surface decreased the number of absorbate molecules, thereby decreasing the charged-impurity scattering in MoS_2 and charge traps.[1] First, the MoS_2 flakes were identified and characterized under an optical microscope using variations in contrast. Figure S1a is a typical optical microscopy image of the MoS_2 sample after deposition of the electrode. Figure S1b is the photoluminescence (PL) spectrum of a typical monolayer and bilayer MoS_2 sample that were fabricated on OTS-functionalized substrates, showing the exciton peaks A and B. Due to the difference in the quantum efficiency, the intensity of the exciton peaks A and B in the monolayer MoS_2 are larger than those in the bilayer MoS_2 samples. Another PL peak (I) at $\simeq$1.6 eV corresponding to the indirect interband transition was observed in bilayer MoS_2. Figure S1c shows a Raman spectrum (blue curve) of a monolayer MoS_2 sample with two characteristic peaks at 388.7 cm^{-1} and 407.0 cm^{-1} that correspond to the E_{2g} and A_{1g} resonance modes. The difference between the two peaks is $\simeq$18.3 cm^{-1}, which is consistent with that obtained for the monolayer MoS_2 from previous reports. For comparison, the Raman spectra of the bilayer and trilayer MoS_2 samples are also shown.

We adopted resist-free fabrication to prevent contamination of the MoS_2 samples from the resist residue of the conventional lithography process. We used nanowire as a shadow mask to deposit metallic contacts (Au, 50 nm) with an electron-beam evaporator at a base pressure of $\simeq$ 1.0×10^{-7} Torr. The MoS_2 channel length was $\simeq$1 μm. The MoS_2 devices were then transferred into a cryostat (Janis Research Company, ST-500) for electrical and optical characterization. The samples were stored under a high vacuum of 1×10^{-6} Torr to minimize the undesirable adsorption of chemical substances. A Keithley 237 was used to perform the DC electrical measurements, and a

Keithley 2400 was used to apply the back gate voltage. Figure S1d shows the source-drain current (I_{DS}) as a function of the source-drain bias (V_{DS}), which is linear over the small bias region.

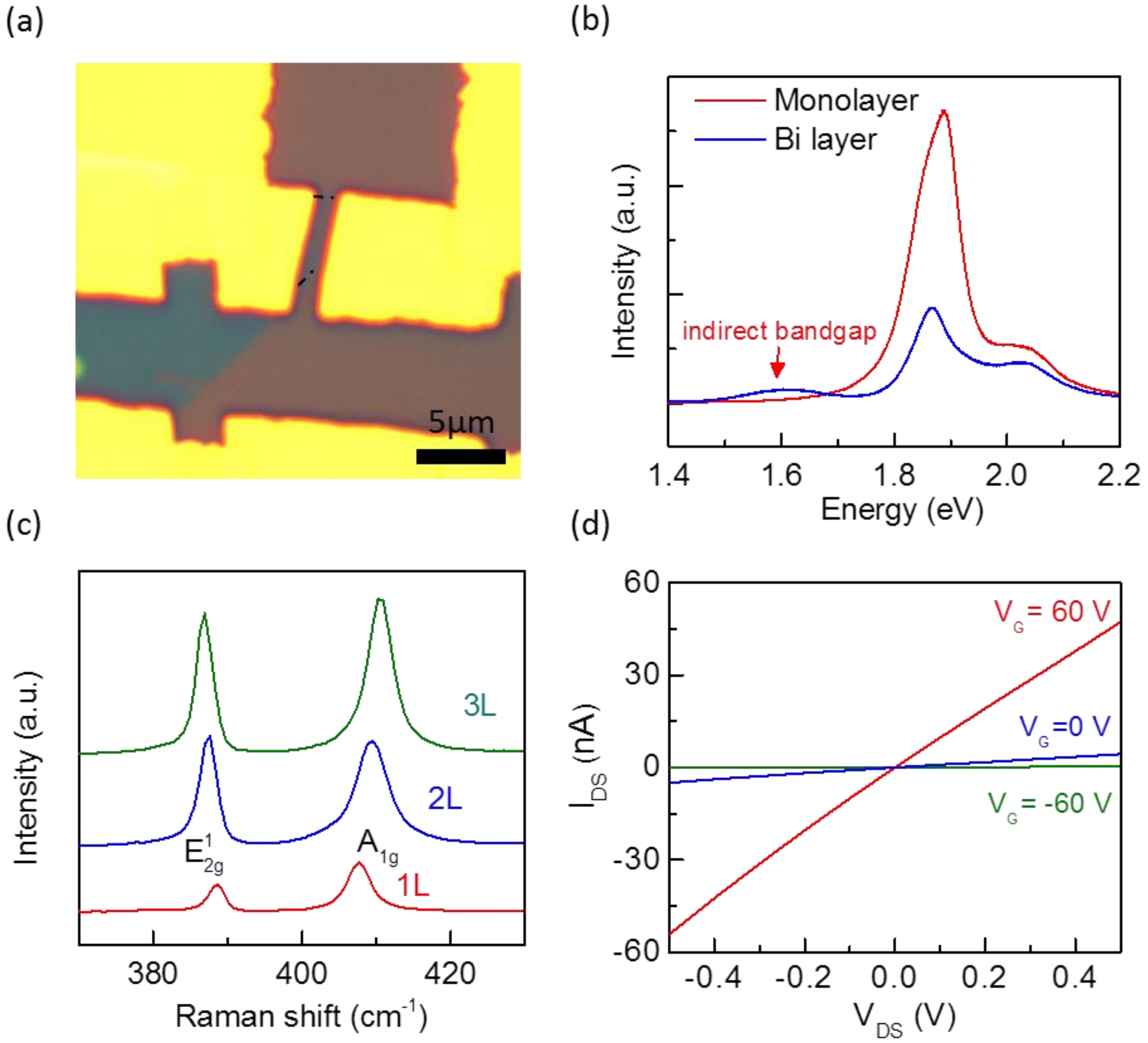

Figure S1. (a) Optical image of MoS_2/OTS FET devices (sample A) discussed in the main text. The channel length is ≃1 μm. (b) PL spectra of monolayer and bilayer MoS_2 on OTS-functionalized substrates. (c) Raman spectra of monolayer, bilayer, and trilayer MoS_2 on OTS-functionalized substrates, showing E_{2g} and A_{1g} peaks of different MoS_2 layers. (d) The $I_{DS}-V_{DS}$ curve of the MoS_2/OTS/SiO_2 device.

S2. Gas Adsorbate Effect

We stored the MoS_2 samples in vacuum (1×10^{-6} Torr) for 12 hours before conducting the measurements to minimize the gas adsorbate effect. Figure S2 shows the transconductance curves of the MoS_2 samples as a function of the back-gate voltage ($G-V_G$ curves). We investigated the effect of the gas adsorbates by comparing the $G-V_G$ curves of the as-fabricated sample under ambient conditions and of the sample that was stored in a vacuum. Under ambient conditions, the MoS_2 device exhibited typical n-type channel characteristics with a mobility of 0.03 $cm^2/V\cdot s$ and an on/off ratio of $\simeq 500$. There was no significant difference between the $G-V_G$ curves before and after irradiation although a small PPC effect appeared (decay time < 10 sec). However, after illumination in a vacuum, the device showed strong persistency in the high-conductivity state for several minutes to hours. Moreover, after storing the sample in a vacuum for 12 hours, the dark current stabilized and was reversible after several cycles of illumination and PPC relaxation. The conductance increased significantly beyond its original level under ambient conditions. Previous studies have shown that the degradation in the electric performance of monolayer MoS_2, which was measured under ambient conditions, can be attributed to adsorbed water and oxygen molecules that act as extrinsic scattering centers.[1-4] By illumination, the gas adsorbates can be removed and pumped out in vacuum, thereby enhancing the electrical characteristics of MoS_2 devices.[5]

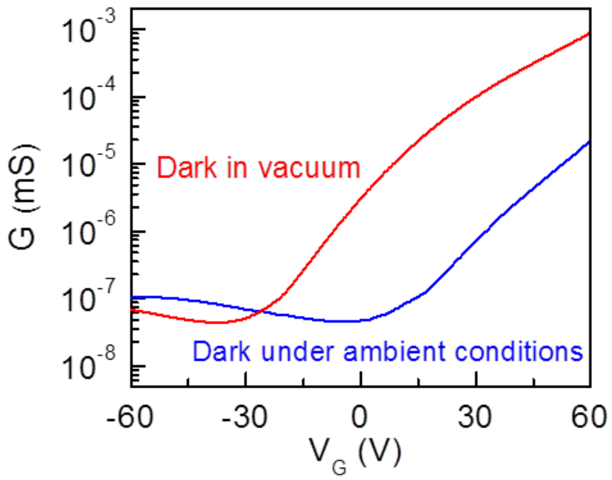

Figure S2. Transconductance of the monolayer MoS_2/OTS/SiO_2 device under ambient and vacuum conditions. Following illumination in vacuum, the enhanced mobility, the on/off ratio, and the threshold voltage are observed.

S3. Photoresponse measurements

Figure S3 shows a schematic of the measurement system that integrated optical microscopy, Raman/PL spectroscopy, and the photoresponse measurements. A solid-state CW laser (Nd:YAG, 532 nm) was employed as the light source in the photoresponse measurements. The incident light was guided into the microscope and focused by an objective (10X, NA 0.3) with a spot size of $\simeq 1.5$ μm. The power density on the sample was estimated at $\simeq 5.6 \times 10^4$ W/cm^2 for an illumination power of 1 mW. For Raman and PL spectroscopy, a 100X objective (NA 0.6) was used with a spot size of $\simeq 0.7$ μm. A mercury-xenon lamp (Hamamatsu Photonics, 150 W) was used as the broadband white light source for the excitation energy dependence of photoresponse (see Figures 3c and 3d in the main text). The white light was selected using a band-pass filter for peak wavelengths from 450 nm to 850 nm (spacing: 50 nm; FWHM: 40 nm). The dependence of the photoresponse on the excitation energy was measured by fixing the power density at 15.9 W/cm^2 for different excitation energies.

We characterized the PPC relaxation by choosing the starting point of the PPC (I_0 in Figure 1b of main text) as the first data point after the photocurrent sharply dropped. Here, we assumed that the band-to-band recombination time was comparable to the lifetime of the PL ($\simeq$100 ps).[6,7] Because the time delay of the PC measurement was 200 ms, which was much larger than the photocurrent relaxation due to band-to-band recombination, the photocurrent after I_0 was dominated by PPC.

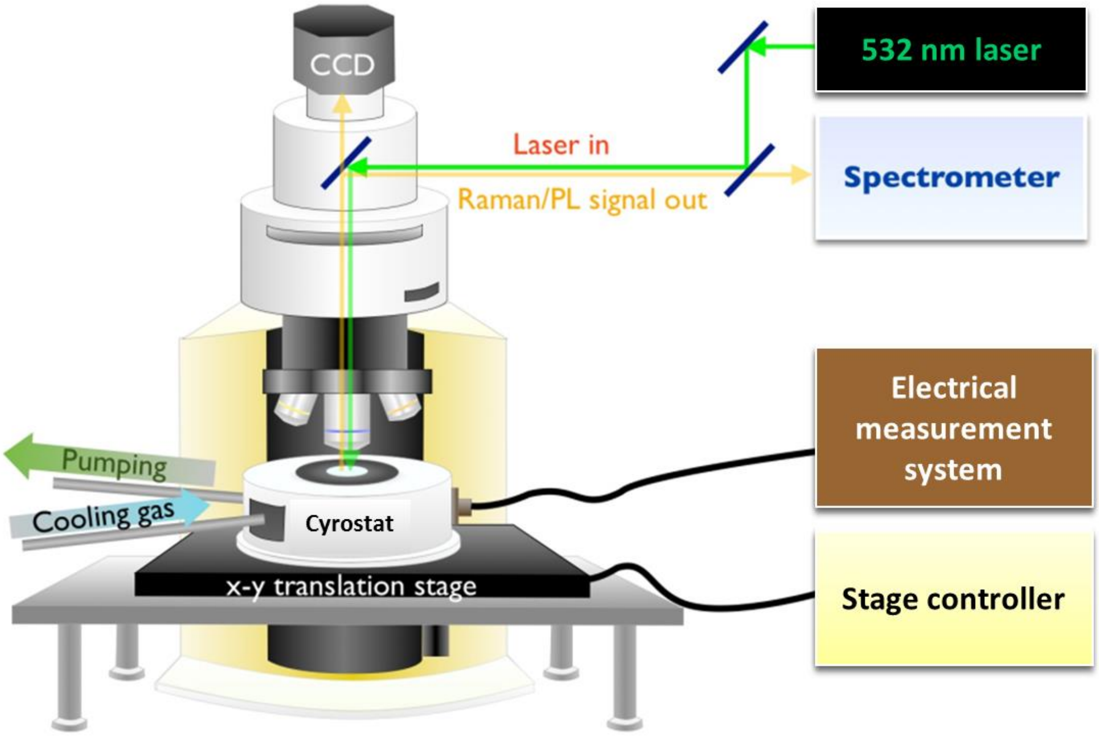

Figure S3. Schematic of the cryostat system with integrated OM, PL, Raman, and photocurrent measurements.

S4. Temperature dependence of PPC relaxation

Before each measurement, the MoS_2 samples were warmed up to room temperature to ensure that the dark current had reached equilibrium and then cooled down in the dark to the desired temperature. Detailed data on the temperature dependence of PPC are shown here, in which the PPC is normalized by I_0 for purposes of comparison. The PPC clearly weakened as the temperature decreased for samples A and B. As shown in Figure 2b in the main text, both τ and β decreased as the temperature decreased. At low temperatures, the PPC dropped more quickly for $t < \tau$ but became more persistent for $t > \tau$, which could be accurately described by a stretched exponential function with a small β.[8]

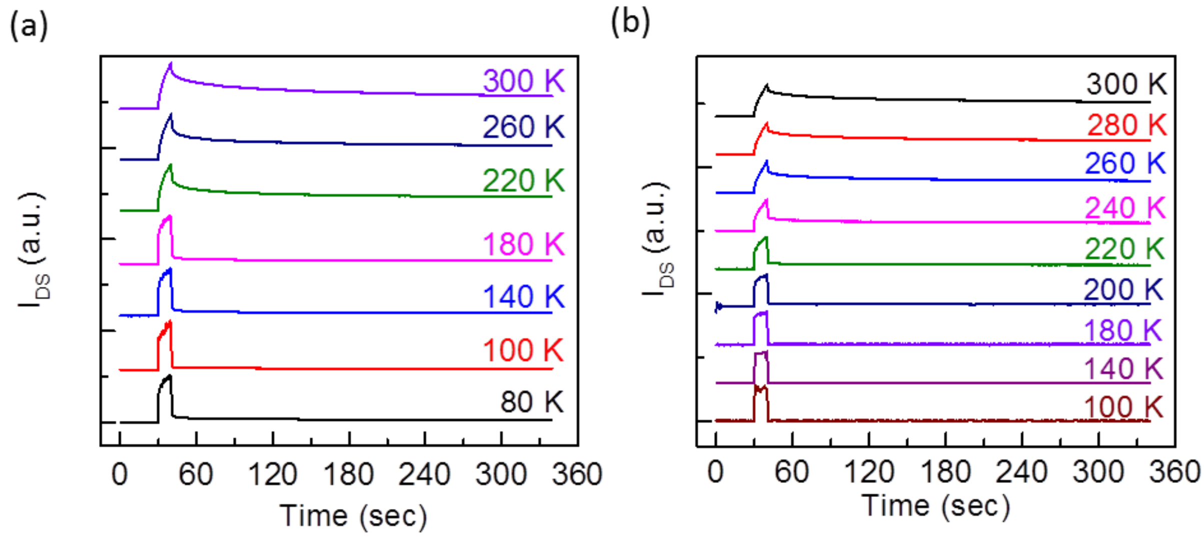

Figure S4. Temporal evolution of the photoresponse for (a) sample A and (b) sample B at different temperatures. The PPC is normalized by I_0 and offset vertically for purposes of comparison.

S5. Fitting of the PPC relaxation curves

To verify the validity of the stretched exponential decay for describing the observed PPC in monolayer MoS_2, we consider other possible relaxation approaches, including single exponential, double exponential, and logarithmic decay. The fitting results of two representative PPC relaxation curves at T = 300 K and 180 K by these different schemes are shown in Figure S5. It can be seen that only the stretched exponential decay yields satisfactory fitting result for the whole temporal range at different temperatures. As mentioned in the main text, the observed stretched exponential decay of the PPC relaxation in our MoS_2 devices suggests a disordered system, which is consistent with the percolation transport model discussed in Figure 5 of the main text.

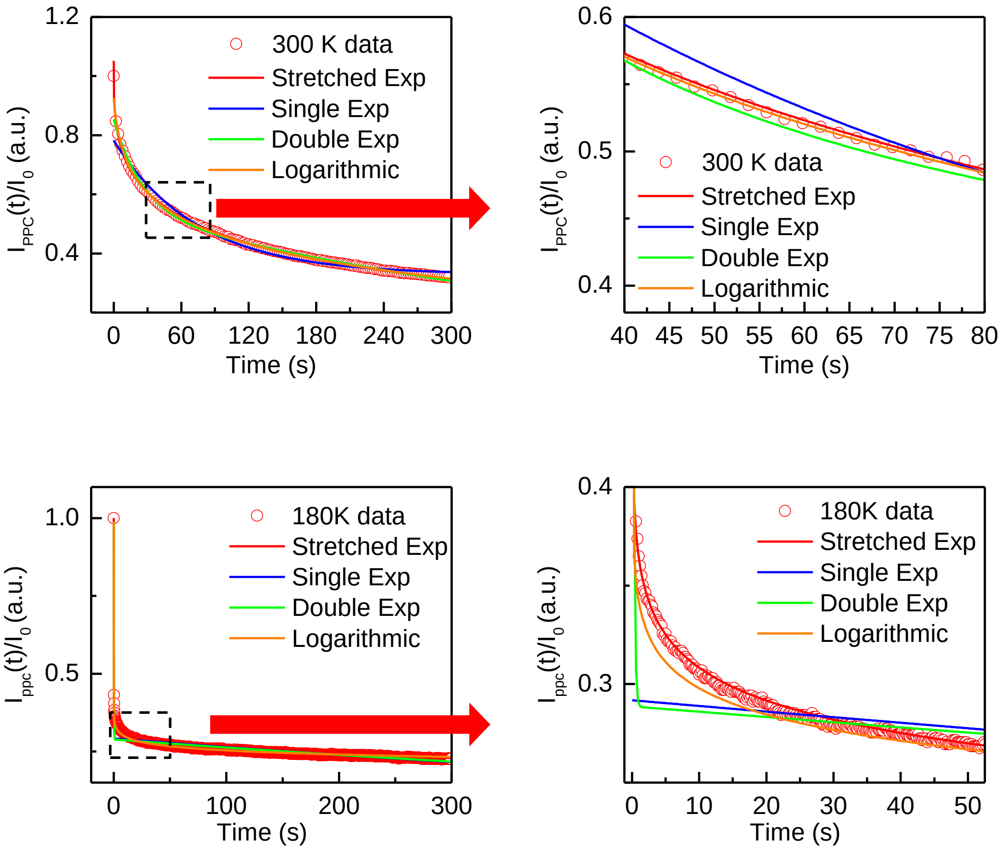

Figure S5. Two representative PPC relaxation curves at T = 300 K and 180 K and the fitting results with various schemes, including stretched exponential, single exponential, double exponential and logarithmic decay.

S6. Gate voltage dependence of the PPC

We discuss the back gate voltage (V_G) dependence of the PPC relaxation here. We show the PPC relaxation for $V_G = -60$ V and $V_G = +60$ V in Figure S6a (also in Figure 4b of the main text), as well as the V_G dependence of τ in Figure S6b. The position of Fermi level, which is controlled by V_G, can greatly affect the carrier density and consequently the conductance in the MoS_2 channel. This V_G dependence of conductance is revealed in Figure 1a of the main text and the related discussion. Nevertheless, the PPC relaxation is mainly determined by the density of the trap states and the extent of the carrier trapping in the RLPF model. Because there are various possible sources of trap states, including electron traps, hole traps, and mid-gap states in MoS_2 samples,[9] it is plausible that the strength of the PPC is correlated to the density of the trap states as Fermi level is tuned with V_G. However, we note that the correlation may be complicated and further study is required to elucidate detailed V_G dependence of the PPC effect.

(a) (b)

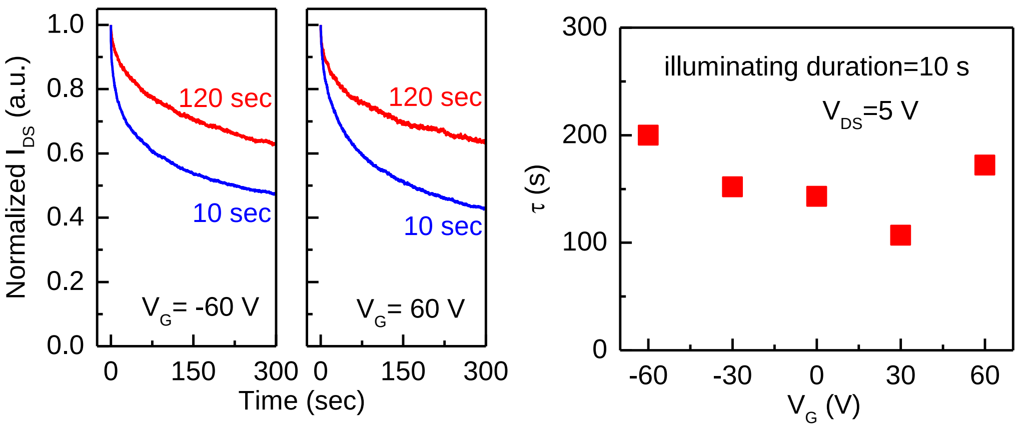

Figure S6. (a) The normalized PPC relaxation curves at $V_{DS} = 50$ mV under $V_G = -60$ V (left panel) and $V_G = 60$ V (right panel). Two different irradiation duration (10 sec and 120 sec) were used to excite the MoS_2 sample. No significant difference between the PPC relaxation for $V_G = 60$ V and $V_G = -60$ V was observed. (b) The fitted τ versus applied V_G at $V_{DS} = 5$ V for illumination duration of 10 sec.

S7. Transport characteristics of the MoS_2 FET

In addition to characterizing the transport behavior of sample B, as discussed in the main text, we show the transfer curves for sample A at different temperatures in Figure S7a. Figure S7b is Arrhenius plot for the conductance of sample A at different V_G values. The transport behavior of sample A could be classified into three different regimes. (1) For $T > 240$ K, the MoS_2 sample exhibited metallic behavior where σ decreased rapidly with the temperature. Over this temperature range, carriers above the mobility edge dominated the transport, resulting in metallic behavior. (2) For $160 < T < 240$ K, the sample exhibited thermally activated behavior. The carrier transport in this temperature regime could be described by a percolating picture in which conduction occurred via a network of spatially distributed puddles. (3) For $80 < T < 160$ K, the correlation of the puddles decreased because of the decrease in the thermal energy, and the carriers could only conduct by tunneling between localized states. Similar to sample B in the main text, the build-up level of the PPC was closely related to the carrier transport, which exhibited percolation behavior ($\mu = 2.2$) above the transition temperature $T_C = 160$ K, as shown in Figure S7c.

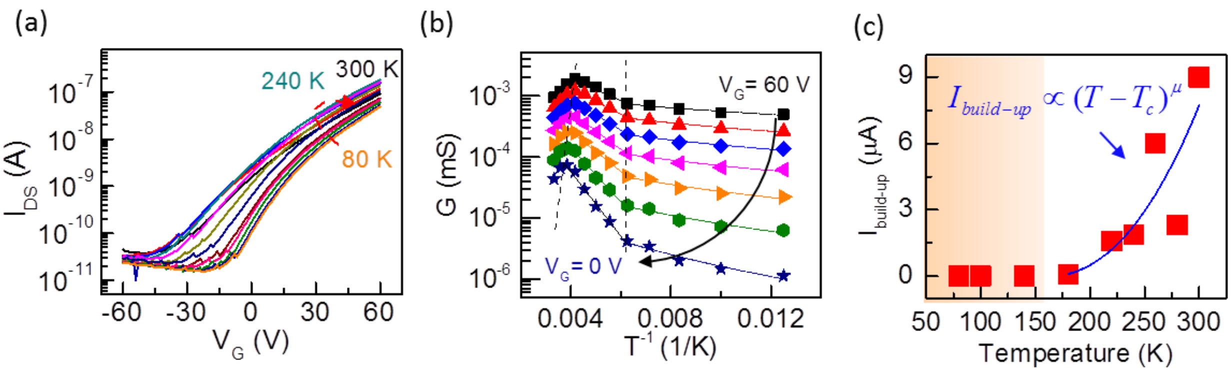

Figure S7. Transport characteristics and PPC for sample A: (a) The two-probe transfer curves at different temperatures. (b) Arrhenius plots of the conductance at different V_G values. (c) The temperature dependence of the PPC buildup level ($I_{build-up}$), where the solid line is the fitted result using the transition temperature $T_C = 160$ K, which is obtained from the transport measurement.

S8. Carrier mobility dependence of the PPC

Figure S8 shows a distribution of τ versus mobility for 7 monolayer MoS_2 samples that were obtained by same illumination condition. The data suggests that the PPC becomes more persistent for higher mobility samples. It is conceivable that more persistent PC is caused by higher density of the trap states in the MoS_2 channel. This could result in shorter average distance between the localized states and greater hopping rate among these states, leading to higher mobility. However, the detail mechanism behind the relation between τ and mobility requires further study.

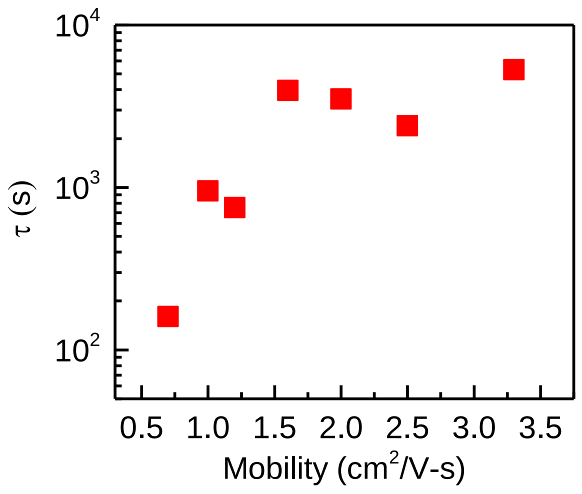

Figure S8. The distribution of τ versus mobility for 7 monolayer MoS_2 samples that were obtained by same illumination condition.

References

1 Li, S. L. *et al.* Thickness-Dependent Interfacial Coulomb Scattering in Atomically Thin Field-Effect Transistors. *Nano Lett.* 13, 3546-3552, doi:Doi 10.1021/Nl4010783 (2013).

2 Tongay, S. *et al.* Broad-Range Modulation of Light Emission in Two-Dimensional Semiconductors by Molecular Physisorption Gating. *Nano Lett.* 13, 2831-2836, doi:10.1021/nl4011172 (2013).

3 Cho, K. *et al.* Electric Stress-Induced Threshold Voltage Instability of Multilayer MoS2 Field Effect Transistors. *Acs Nano* 7, 7751-7758, doi:10.1021/nn402348r (2013).

4 Qiu, H. *et al.* Electrical characterization of back-gated bi-layer MoS2 field-effect transistors and the effect of ambient on their performances. *Appl. Phys. Lett.* 100, 123104, doi:Artn 123104

Doi 10.1063/1.3696045 (2012).

5 Zhang, W. *et al.* High-Gain Phototransistors Based on a CVD MoS2 Monolayer. *Adv. Mater.* 25, 3456-3461, doi:10.1002/adma.201301244 (2013).

6 Korn, T., Heydrich, S., Hirmer, M., Schmutzler, J. & Schuller, C. Low-temperature photocarrier dynamics in monolayer MoS2. *Appl. Phys. Lett.* 99, 102109, doi:Artn 102109

Doi 10.1063/1.3636402 (2011).

7 Kozawa, D. *et al.* Photocarrier relaxation pathway in two-dimensional semiconducting transition metal dichalcogenides. *Nat Commun* 5, 4543, doi:10.1038/ncomms5543 (2014).

8 Johnston, D. C. Stretched exponential relaxation arising from a continuous sum of exponential decays. *Phys. Rev. B* 74, 184430 (2006).

9 Furchi, M. M., Polyushkin, D. K., Pospischil, A. & Mueller, T. Mechanisms of Photoconductivity in Atomically Thin MoS2. *Nano Lett.* 14, 6165-6170, doi:10.1021/nl502339q (2014).
